# Nurses’ Perspectives and Experiences of Using a Bed-Exit Information System in an Acute Hospital Setting: Mixed Methods Study

**DOI:** 10.2196/64444

**Published:** 2025-02-05

**Authors:** Stefan Walzer, Isabel Schön, Johanna Pfeil, Sam Klemm, Sven Ziegler, Claudia Schmoor, Christophe Kunze

**Affiliations:** 1 Care and Technology Lab Furtwangen University Furtwangen im Schwarzwald Germany; 2 AGP Social Research FIVE e.V. Freiburg Germany; 3 Center of Implementing Nursing Care Innovations Freiburg, Nursing Direction, Medical Center, University of Freiburg Freiburg Germany; 4 Clinical Trials Unit Faculty of Medicine and Medical Center University of Freiburg Freiburg Germany

**Keywords:** cognitive impairment, bed-exit, technology, fall prevention, inpatient, hospital, mixed methods, nurse, information system, acute hospital, support, online questionnaire, cognitively impaired, workload

## Abstract

**Background:**

Technology that detects early when a patient at risk of falling leaves the bed can support nurses in acute care hospitals.

**Objective:**

To develop a better understanding of nurses’ perspectives and experiences with a bed-exit information system (BES) in an acute care hospital setting.

**Methods:**

BES was implemented on 3 wards of a university medical center. Nurses completed 2 online surveys at each time point (P0 and P1) and participated in focus groups before (P0) and after (P1) implementation. Additional patient data were collected. Descriptive statistics summarized the survey results, while content analysis was applied to focus group data. Patient rates and adverse events in both phases were compared using negative binomial models. Reporting of this study adhered to the GRAMMS checklist.

**Results:**

A total of 30 questionnaires were completed at P0 (30/72, 42%) and 24 at P1 (24/71, 33%). Of the participants, 15 completed both questionnaires (complete cases). At P1, 64% (9/14) of participants agreed that their perceived workload and strain in caring for patients with cognitive impairment was reduced by the use of the BES. The adverse event rate per patient per day was reduced by a factor of 0.61 (95% CI 0.393-0.955; *P*=.03). In addition, 11 nurses participated in 4 focus groups before and after the intervention. Participants found it challenging to operationalize the use of the BES due to the heterogeneity of care settings, but certain behaviors of patients with cognitive impairment were recognized as indicating a need for intervention. Negative experiences included information overload and alarm fatigue, leading to occasional removal of the system.

**Conclusions:**

While BES provides some support in managing patients with cognitive impairment, its impact remains limited to specific scenarios and does not significantly reduce nurses’ workload or strain. Our findings highlight the need to manage expectations of BES performance to ensure alignment between expected and actual benefits. To improve BES effectiveness and long-term implementation, future research should consider both objective measures of patient care and subjective factors such as nurse experience, structural conditions, and technical specifications. Improving information mechanisms within call systems could help reduce alarm fatigue and increase perceived usefulness. Overall, successful integration of BES in acute care settings will require close collaboration with nursing staff to drive meaningful healthcare innovation and ensure that the technology meets the needs of both patients and nurses.

**Trial Registration:**

German Register for Clinical Studies DRKS00021720; https://drks.de/search/de/trial/DRKS00021720

## Introduction

### Background

Globally, the number of older adults admitted to hospitals in emergency situations is increasing, and this trend is expected to continue as a result of current demographic changes [1]. Currently, older adults account for approximately two-thirds of all hospital inpatients, and up to 50% of this group show signs of cognitive impairment, including forms associated with dementia [2,3]. Patients with cognitive impairment often find it challenging to adhere to standardized care plans in the hospital setting [1]. They are at increased risk of developing responsive behaviors (responsive behavior is a term, which represents the effect of cognitive impairment on actions, words, and gestures of a person) and continued cognitive and functional decline [4]. Typical behaviors include an inverted day-night cycle or lack of adherence to care plans [5]. We refer to such behaviors as adverse events (AEs) in the context of nursing care when they affect the health or well-being of patients or increase the workload of nurses (see the Adverse Events in the Context of Nursing Care section). A particular challenge for nurses in hospitals is motor agitation in and around the bed, as well as related incidents such as falls or the disconnection of catheters or vascular access [6]. Kang and Hur [7] reported in a qualitative meta-synthesis that these symptoms were associated with a significantly higher workload [7]. In light of this, technological innovations have been discussed as a potential support for nurses and caregivers [8-10]. Bed-exit information systems (BES) serve as warning systems designed to monitor bed exits or patient movements in bed. This should enable nurses to prevent (or respond more quickly to) falls or other related events [11]. Nurses’ perspectives and experiences on a particular technology play a central role in whether it will be successfully used and adopted over time [12]. Understanding and addressing these attitudes is essential to promoting successful and sustained technology adoption in the acute care hospital setting. In this context, little attention has been paid to nurses’ perspectives and experiences on the use of a BES [13,14]. Previous studies have predominantly addressed the efficacy and effectiveness of BES in terms of reducing falls or fall rates [15,16].

### Aim and Research Questions

Based on these findings, the aim of this study was to develop a better understanding of nurses’ experiences, views, and needs regarding BES in an acute care hospital setting.

In terms of the aim, this study addresses the following research questions:

- What are nurses’ perspectives and experiences of using a BES in an acute hospital setting?
- What is the perceived workload and strain of nurses dealing with patients with cognitive impairments and how does it change with the use of a BES?
- What is the number of AEs before and during the use of a BES?

## Methods

Reporting of the study followed the Good Reporting of a Mixed Methods Study (GRAMMS) criteria proposed by O’Cathain et al [17].

### Design and Setting

**Figure 1:**
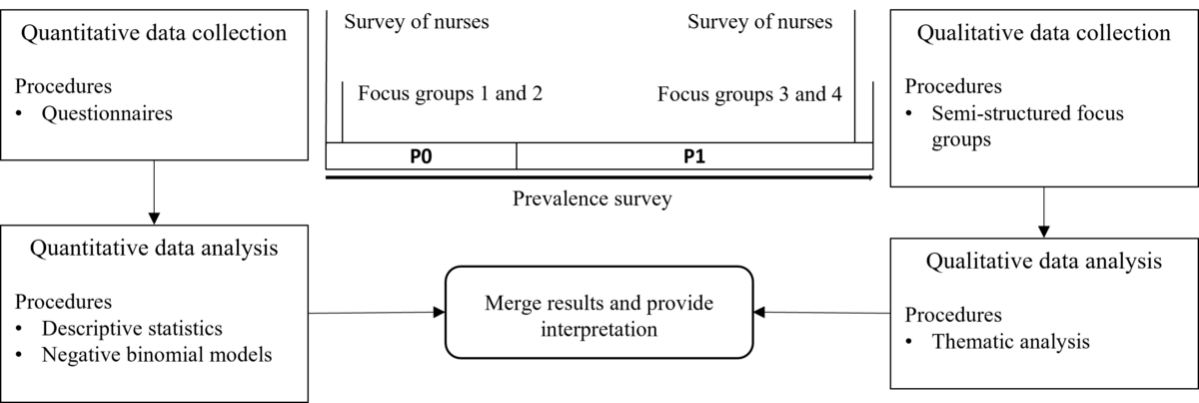
Study design.

A parallel mixed methods triangulation design (Figure 1) with quantitative surveys and qualitative focus groups (FGs) was used to gain more insight from multiple perspectives [18]. A fundamental premise of mixed methods research is that the merging of quantitative and qualitative data provides a more complete understanding of the research question than using either data source in isolation. As defined by Creswell [19], mixed methods research is “research in which the investigator collects both quantitative (closed) and qualitative (open) data, integrates the 2, and then draws interpretations based on the strengths of both data sets to understand the research problem” [19]. Integration of methods occurred in the discussion chapter of the study, where the authors collaboratively interpreted and consolidated the results. The study was conducted in 3 wards assigned to the departments of neurosurgery, surgery, and radiology at a university medical center in southern Germany with 2 phases of data collection over a total period of 190 days. The first phase without the use of BES (P0) lasted 64 days. In the second phase (P1), a BES was available for 126 days as a tool to assist nurses in caring for patients with cognitive impairment. During the study period, 912 adult patients were treated in the first phase of the study (P0). In the second phase (P1), 1748 patients were treated.

#### Bed-Exit Information Systems

After a rigorous, multistage selection process, the research group chose a sensor mat in the form of a mattress topper from a Swiss manufacturer. By lowering the body weight on the sensors, the device transmits information about (potential) bed exits to the call system through a radio transmitter. The manufacturer has developed this technology specifically for people with cognitive impairments such as delirium, dementia, and sleep disorders. The sensor mat, equipped with a radio transmitter, was placed under the bed mattress and the corresponding radio receiver was connected to the call system. The call system of the participating wards was not able to distinguish between alarms triggered by the sensor mat and other calls. In this study, the assessment of patient cognitive impairment was performed by nurses. The decision to use the BES was also made independently by the nurses, as not all patients with cognitive impairment exhibit symptoms such as motor agitation. Training sessions were conducted for all wards, focusing on the handling and use of the equipment according to the manufacturer’s intended scenarios. These sessions were held on separate days during the handover period from the early to the late shift. If additional training was deemed necessary, additional training sessions could have been scheduled. One of the authors conducted the training sessions.

#### Patients With Cognitive Impairment

For this study, cognitive impairment is defined as temporary or persistent problems with mental performance, regardless of age. Typical complaints include increasing forgetfulness, decreased attention, difficulty concentrating, speech problems, disorientation, or memory loss. Cognitive impairment can occur after surgery, as part of an illness, or as a result of general age-related decline [20].

#### Adverse Events in the Context of Nursing Care

Responsive behaviors can lead to events that (1) disrupt acute inpatient care and can directly or indirectly endanger the affected patient or fellow patients and (2) are related to the unintentional (potential) leaving of the bed without supervision [21]. As part of the prevalence survey, the number of the following AEs was recorded: (1) finding a patient out of bed; (2) falling of a patient; (3) finding a patient in a helpless situation; (4) disconnection or dislocation of accesses, drains, or catheters; (5) use of restraints; (6) leaving the ward or the hospital unsupervised, and (7) disturbing or endangering of fellow patients. These AEs were previously identified as particularly important in 2 workshops with nurses at the university hospital, which is consistent with recent studies [1,22].

### Participants

#### Overview

The surveys and FGs were conducted among nurses who met the inclusion criteria. The target group consisted of all nurses who met the predefined criteria listed in Textbox 1, while individuals who did not meet these criteria were excluded. At P0, the total number of registered nurses used in all wards was 72, which decreased to 70 at P1. Potential participants were informed about the surveys and the FGs during ward meetings and by email using internal institutional mailing lists and were invited to participate. Interested nurses received detailed written information about the study and what participation entailed (see the Ethical Considerations section for further details). The surveys and FGs were completed during working hours with the permission of the respective organization, and no additional incentives or rewards were offered to participants.

**Inclusion and exclusion criteria.**

**Inclusion criteria**

- Age ≥18 years.
- Nurses with at least 3 years of apprenticeship or equivalent international training with professional recognition in Germany.
- Employees working on the included wards during the study period.

**Exclusion criteria**

- Employees of the included wards who belong to other professional groups.
- Employees of the reserve pool.
- Nurses from other wards helping out.
- Trainees.

#### Data Collection

The study followed a parallel mixed methods approach with the following elements:

1. Surveys and FGs of nurses at time points P0 and P1, before and after BES use
2. A prevalence survey on the number of patients with cognitive impairment and AEs related to their leaving the bed throughout the study period (P0/P1) and, in the intervention phase (P1), the number of patients with cognitive impairment who received the BES.

#### Surveys of Nurses

The surveys were conducted using a standardized questionnaire based on 2 questionnaires [23,24], supplemented by specific items related to the setting and technology. A total of 22 (P0) and 27 (P1) items assessed respondents’ perceived strain in dealing with patients with cognitive impairment, expectations (P0) and experiences (P1) of the BES, and sociodemographic data. Items in the perceived strain category focused on AEs and the care process, which were associated with high levels of potential strain for nurses in the Isfort et al [23] study. With the exception of 2 unipolar-scaled items assessing general attitudes and acceptance of IT, items were expressed on a bipolar 7-point Likert scale.

#### Focus Groups

The 4 FGs were conducted by 2 moderators (SW and JP). Both moderators involved are experienced qualitative researchers, and one moderator (SW) is a registered nurse. FGs are a special type of group interview in which a moderator leads a discussion focused on a specific topic [25]. FGs are useful for obtaining information about beliefs, attitudes, thoughts, and perceptions about a particular topic from several people in a short period of time. The interaction of the participants within the group plays an important role in the generation of data [26]. A semistructured interview guide was used to structure the discussion. This guide was developed according to the recommendations of Kallio et al [27]. The structure and content of the guide are based on the current research findings mentioned above. A semistructured interview guide was chosen because it provided a framework for exploring the key concepts of interest while allowing for flexibility and openness in the discussion. During the development phase, several initial versions of the interview guide underwent multiple internal reviews and modifications by all members of the research team. Nurses were asked about their perspectives (P0) and experiences (P1), as well as barriers and opportunities for sustainable use of the BES, and the discussions were recorded and transcribed.

#### Prevalence Survey of Patients and Adverse Events

The prevalence survey was conducted retrospectively once a day for the last 24 hours by the nurses of the 3 wards. The number of patients with cognitive impairment and a tendency to get out of bed (first and second phases), the number of patients using a system (second phase), and the number and type of AEs that occurred in patients (first and second phases) were recorded anonymously on a documentation sheet (occupancy and fall prevalence statistics from the medical center’s medical controlling and reporting systems were used as a reference for the quantitative data).

### Analysis

#### Quantitative Data

The Clinical Trials Unit at the University Medical Center analyzed the quantitative data. Intraindividual changes in nurses’ responses between phases were presented. AE rates of both phases were compared using negative binomial models [28]. Three different analyses were performed: (1) a raw overall comparison of both phases without considering individual wards, (2) a comparison of both phases separately for the 3 wards, and (3) an overall comparison of both phases adjusted for differences between wards. AE rates and multiplicative phase comparisons (ratio of AE rates in P1 vs P0) were obtained from the negative binomial models with 95% CIs, and tests of the hypothesis of equality of AE rates in P0 and P1 were performed at a 2-sided 5% significance level.

#### Qualitative Data

Content analysis was applied using a deductive-inductive approach [29]. The transcripts were read by 3 members of the research team (SW, IS, and JP) to familiarize themselves with the data. Codes were created through condensation and then abstracted into categories. The coders (SW and JP) reviewed the codes and categories, discussed any discrepancies, and made revisions. Finally, narrative summaries were written for each category, and thematic descriptions for the main categories were developed based on these summaries.

#### Setting

The study was conducted at the University Medical Center Freiburg.

### Ethical Considerations

Approval was obtained from the Ethics Committee of the University of Freiburg (106/20 [MPG §23b]) and the Staff Council. Before the start of the study, all potential participants were provided with a comprehensive written document containing information about the study, including its purpose, objectives, procedures, and data protection measures. Participants were informed of the decision of the responsible committees and were given the opportunity to contact the investigators with any questions throughout the study. For participants who completed the surveys, they were explicitly informed at the beginning of the questionnaire that by submitting their responses they were indicating that they had read and understood the enclosed information letter and were giving their informed consent to participate in the survey. In the case of the FG, written informed consent was obtained from each participant at the beginning of each group session, confirming that they had been informed of the study details and were willing to participate in the study. Participation in the surveys and FGs was voluntary, no personal information was collected, and anonymity (surveys) or pseudonymization (FGs) was always maintained.

## Results

### Results of the Surveys

In total, 30 questionnaires were completed at P0 (30/72, 42%) and 24 at P1 (24/71, 33%). Among the total participants, 15 participants completed both questionnaires (complete cases). The sociodemographic characteristics of the participants are shown in Table 1. Approximately two-thirds of the respondents had at least 15 years of general nursing experience and the same amount of experience working with patients with cognitive impairment.

**Table 1:** Sociodemographic characteristics.

Characteristics		Before implementation (P0; n=30), n (%)	After implementation (P1; n=24), n (%)
**Gender**			
	Women	20 (67)	12 (50)
	Men	10 (33)	10 (42)
**Age (years)**			
	≤40	10 (33)	9 (38)
	>40	20 (67)	14 (58)
**General work experience (years)**			
	≤15	8 (27)	6 (25)
	>15	22 (73)	16 (67)
**Work experience with patients with cognitive impairment (years)**			
	≤15	10 (33)	8 (33)
	>15	20 (67)	15 (63)

The following results refer to the complete cases to allow a comparative view.

#### General Attitude Toward (Information) Technologies

The survey conducted at P0 showed that nurses generally had a positive attitude toward IT. This was reflected in the finding that 90% (27/30) of respondents recognized the benefits of IT, agreeing that it offered significant benefits and considering it essential in today's world.

Despite this overall positive outlook, some nurses expressed concerns and fears about using such technology. Overall, 14% (4/30) admitted that they feared making irreversible mistakes when using technology. In addition, 14% (4/30) found most technology-related issues challenging and 7% (2/30) said they were frightened by the possibility of using technology they had never worked with before.

The following results refer to the complete cases to allow a comparative view. Multimedia Appendix 1 shows that the participants rated their experience in dealing with IT or acceptance of IT rather positively (experience: median 7 (IQR 6-8); acceptance: median 8 (IQR 7-9).

#### Perceived Strain

**Figure 2:**
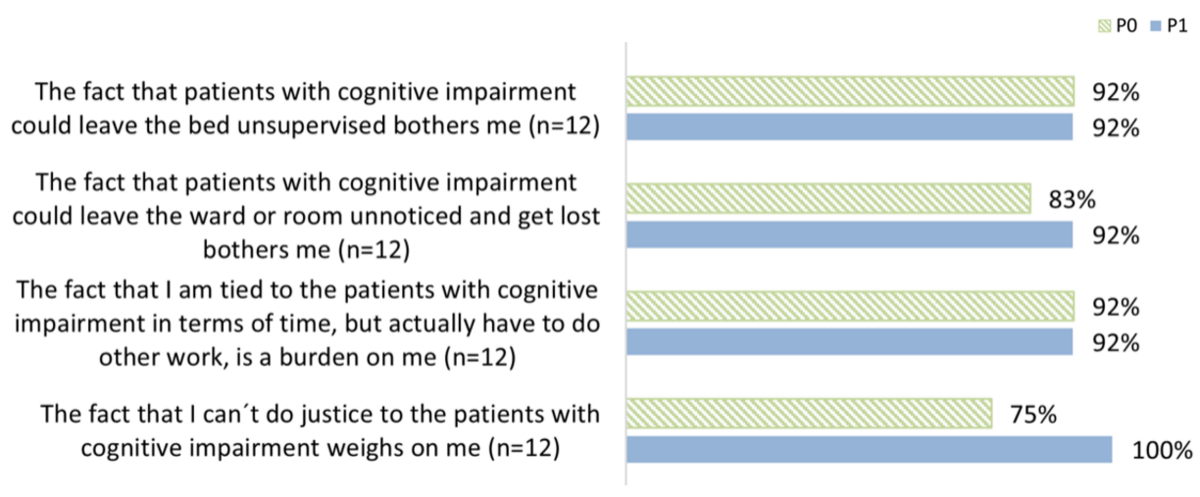
Perceived strain (ranging from moderate to very high).

The vast majority of participating nurses felt moderately to very strongly strained by patients with cognitive impairment, both before and after the use of BES (Figure 2). For example, 75% (9/12) in P0 and 100% (12/12) in P1 of the participating nurses felt strained by not being able to do right by the patients, and 92% (11/12) in P0 and P1 felt strained by the fact that patients with cognitive impairment could leave the bed unattended.

#### Expectations and Experiences Regarding the Use of Bed-Exit Information Systems

**Figure 3:**
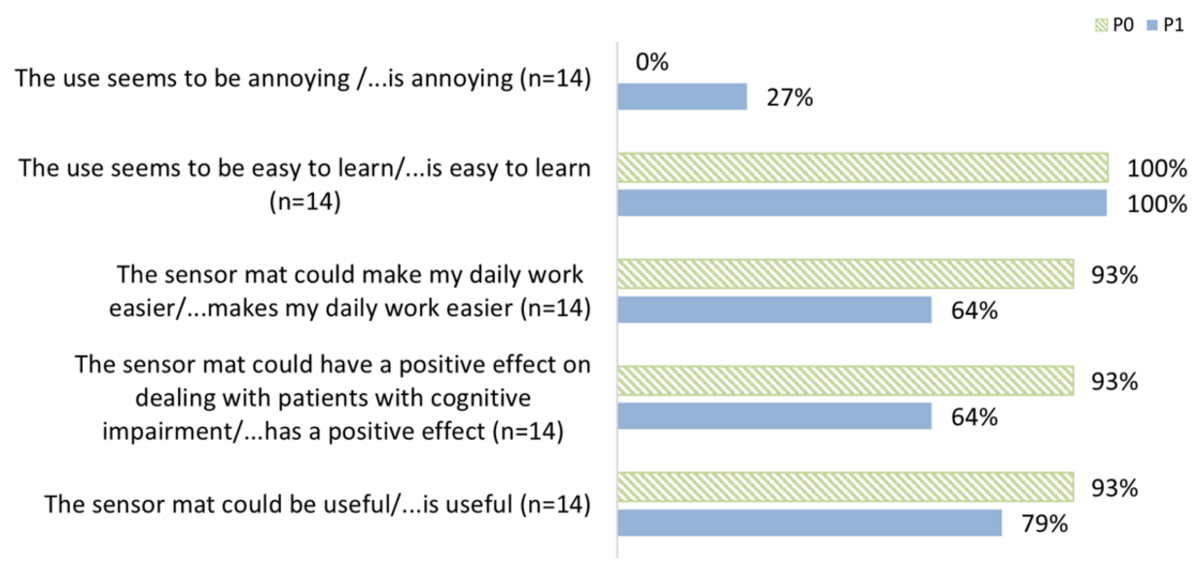
Expectations and experiences regarding the use of technology (statements ranging from somewhat agree to strongly agree).

Among the nurses who participated in both surveys, their expectations regarding the use of BES did not match their experiences in most categories (Figure 3). For example, after the intervention, 64% (93%, P0) agreed that BES can have a positive effect on the management of patients with cognitive impairment and that daily work becomes easier, and 79% (93%, P0) expected that BES could be useful.

### Results of the Prevalence Survey on Adverse Events

The collection of data through the questionnaires took place around 2 PM and referred to the period of the previous 24 hours. Analysis of the prevalence survey showed that in P0, patients with cognitive impairment were treated on 78% of the survey days (199/256). In P1, patients with cognitive impairment were treated on 60% of survey days (302/504), and BES was used by nurses on 20% of survey days (103 of 504). On 79 of 199 days in P0 (40%), 222 AEs were recorded in patients. In P1, 253 AEs were recorded on 82 of 302 days (27%).

**Figure 4:**
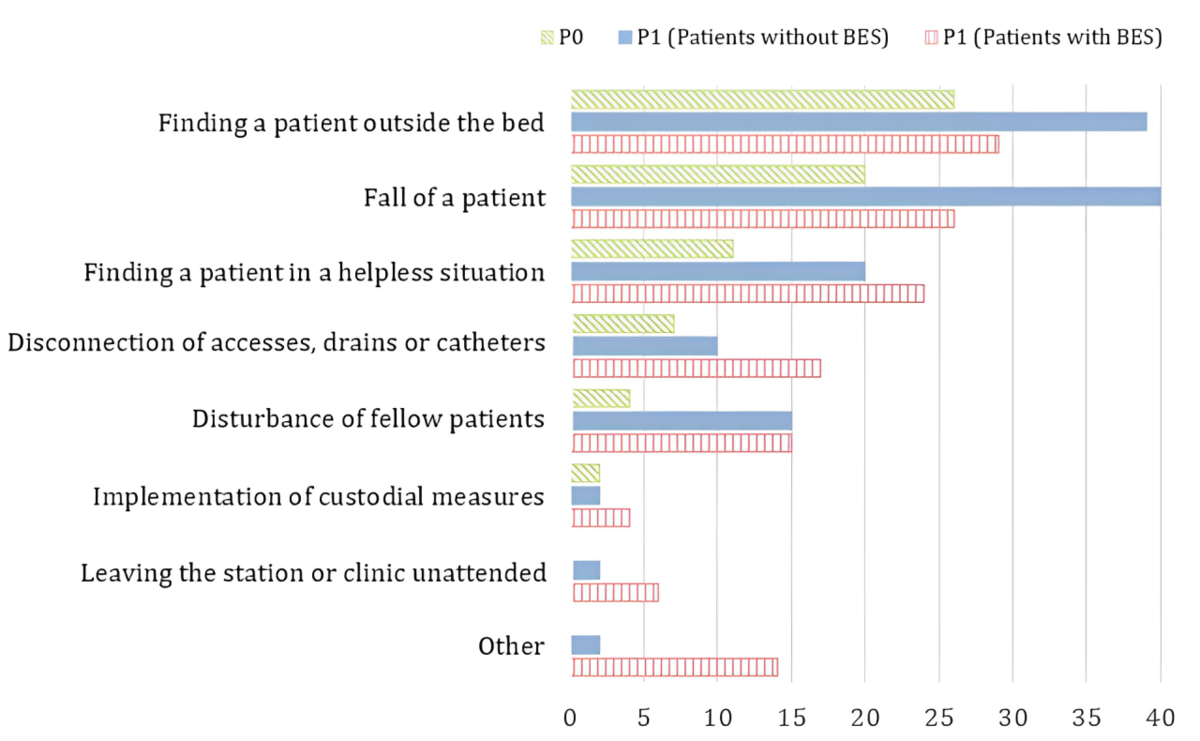
Adverse events (type and number).

The visualization in Figure 4 illustrates the occurrence of each defined AE. For each day, whether an event occurred at least once or not was recorded. It is important to note that the frequencies of the 3 categories presented can only be effectively compared within their respective contexts, as both the duration and the number of patients for each category vary. The most frequent event is the discovery of patients out of bed. This event may be due to a previous fall that left the patient in a vulnerable state. Conversely, a comparatively rare but meaningful event involves patients leaving the ward or clinic unattended, posing a potential risk of disorientation and “getting lost.”

The average AE rates per patient per day in P0 and P1 and the ratio of AE rates in P1 versus P0 with 95% confidence intervals from the raw overall comparison without considering the individual wards shown in Table 2.

**Table 2:** Adverse events in the context of nursing care rates per patient and day in P0 and P1.

Phase	AE^a^ rate (95% CI)	*P* value
0	0.49 (0.36-0.67)	—^b^
1	0.35 (0.27-0.46)	—
1 vs 0	0.73 (0.48-1.09)	.12

^a^AE: adverse event.

^b^Not applicable.

Table 3 provides a comparison of AE rates between P0 and P1 separately for the three wards.

**Table 3:** Adverse event rates per patient per day with interaction between phase and ward.

Ward	Phase	AE^a^ rate (95% CI)	*P* value
1	0	0.29 (0.17-0.49)	—^b^
1	1	0.57 (0.39-0.82)	—
1	1 vs 0	1.97 (1.03-3.75)	.04
2	0	0.28 (0.13-0.60)	—
2	1	0.26 (0.08-0.08)	—
2	1 vs 0	0.92 (0.23-3.70)	.91
3	0	0.70 (0.47-1.05)	—
3	1	0.20 (0.13-0.29)	—
3	1 vs 0	0.28 (0.16-0.50)	<.001

^a^AE: adverse event; in the context of nursing care.

^b^Not applicable.

The picture was different for each ward. While ward 1 had a relatively low AE rate of 0.29 (95% CI 0.17-0.49) in P0 and increased by a factor of 1.97 (95% CI 1.03-3.75) in P1, ward 2 showed similarly low AE rates in P0 and P1. In ward 3 with 125 patient days in P0 and 248 patient days in P1, the relatively high AE rate in P0 decreased from 0.70 (95% CI 0.47-1.05) in P1 to 0.20 (95% CI 0.13-0.29). The statistical test for interaction between phase and ward showed a *P* value of .00. The overall comparison of AE rates between P0 and P1, adjusted for differences between the wards, is shown in Table 4.

**Table 4:** Adjusted adverse event rates per patient per day in P0 and P1.

Phase	AE^a^ rate (95% CI)	*P* value
0	0.47 (0.33-0.67)	—^b^
1	0.29 (0.21-0.41)	—
1 vs 0	0.61 (0.39-0.95)	.03

^a^AE: adverse event; in the context of nursing care.

^b^Not applicable.

Thus, in a summary comparison of AE rates per cognitively impaired patient per day on each ward, there was a reduction in the AE rate by a factor of 0.613 (95% CI 0.393-0.955; *P*=.03).

### Results of the FGs

A total of 11 nurses participated in the FG*s*. Two FGs with similar questions were held before (P0) and 2 were held after the use of the BES (P1). Each discussion lasted for 2 hours. The group size varied from 2 (see limitations for more details) to 5 participants, and 3 nurses participated in both P0 and P1. The key findings from the thematic descriptions are presented: (1) challenges in the use of BES and (2) opportunities in the use of BES; once from an expectation perspective (P0) and once from an experience perspective (P1).

#### Challenges

The nurses who participated in the preintervention FG identified several challenges in using BES. In the hospital setting, the exact causes of AEs are often not (or not fully) understood (also due to lack of biographical information). Therefore, concrete scenarios for the use of BES remain vague in advance. Consequently, the most specific expectations relate to the institutional framework: it is assumed that the system can be used in cases where continuous monitoring is needed, but no constant observers (sitters) and no other strategies (such as transfer to the intensive care unit) are available. Of course, nurses have also developed strategies to deal with AE independently of technical aids. Nontechnical alarms and safeguards such as mattresses on the floor in front of beds or plastic cups on door handles are used. However, the desire for sitters, who participants felt could best counteract AE, is mentioned very often.

[…] so, I see in general such a mat, a mat for movements ... I think, that [the BES] is the wrong way, because we actually need more people in the clinic […]

P0_FG1

After using the BES (P1), the participants confirmed that the diversity of conditions and symptoms makes operationalization a challenge. It became clear that the question, “Who gets a BES and for what reasons?” is not easy to answer. The heterogeneity of care situations also results in a variety of situations that are challenging for caregivers. Nevertheless, it was mostly specific behaviors of the patients with cognitive impairment that indicated intervention. One nurse summarized this as follows:

The cognitively altered [people], who are too mobile for the bed rail to protect [them]. [...] If this is someone who can perhaps still move a foot or even only half of it, he doesn’t necessarily need a sensor mat, because he can’t stand up anyway, yes. But [the one who is] just cognitively impaired, still very lively, very mobile, agile: he gets a mat.

P1_FG1

The discussions also revealed negative experiences with BES. Depending on the workload of the nurses, it became clear that the frequency of information could lead to delays and additional workload. After an initial rush to get information from the BES, information or alarm fatigue set in due to the frequency of information, and the systems were sometimes removed because nurses saw more disadvantages than benefits.

[…] *They said: ‘Oh, that’ll be the mat again.’ But I mean - I think you still have to look. You can’t say: ‘My God, it’s just the mat.’ And just leave it there.* [P1_FG1]

#### Opportunities

Before using the BES (P0), the nurses discussed the need for more staff and sitters as mentioned earlier. Since sitters are not always available, despite the preference for sitters, a technological device such as the BES is seen as an alternative, especially at night.

And then we said: ‘well better now than never’ - sounds interesting and if it [BES] is of any use something […] then we’ll cope with that. Because, I think for us, at night or also during the day depending on / - because sitters, that is another problem, you don’t always get them and if this [BES] is of any help: great.

P0_FG1

As intended by the manufacturer, the BES is used for wandering patients. However, the participating nurses differentiated between whether the person felt a “harmless” urge to move, for example, to walk down the hallway, or whether there was an acute risk of falling. Participants indicated that BES was useful when patients with cognitive impairment were at risk of being lost if they left the building quickly and purposefully. In this context, BES has special potential at night.

*I actually have the feeling that the situation [...] with people who absolutely have to go to the toilet at night and then they can't find the light - they don't even think about the bell****-****the night guards have already benefited from it and they also went into the room more quickly at night because they knew there was a mat in there […].* [P1_FG2]

After using the BES, the participating nurses were mostly positive about the reliability of the alarms and considered it acceptable that moderate technical problems occurred in a few cases, for example, false-positive alarms. From their point of view, the BES is a technical aid that can be used in individual situations, such as pressure mattresses or patient lifts, and as such has its place in everyday care.

I put that in once, plug it in and then it’s there. When I put an infusion pump or an ultrasound or something else into the room, it’s there, too. Maybe I’m too naive now, but of course it fits into the daily nursing routine. Because, as we have now experienced, it does prevent falls in some cases. Of course, it fits in there, for me.

P1_FG1

Even before use, participants identified potential support in the early detection of AE. This can help give them a sense of security and safety. BES can support and relieve nurses by alerting them to certain critical situations. One nurse summarized this as follows:

Then I already know how fast I can run. But that's the good thing, that I actually already know, ok, I don't hear it rumbling and then run, but it rings and I run and it doesn't rumble.

P0_FG1

This potential was also perceived by participants after use. In certain situations, the advantages of the BES may outweigh the disadvantages, such as constant alerting and the associated additional workload.

So, it was little more relaxed. Because you stop-if it rang, then there was something. Then you could go, more or less calmly. Sure, it rang all the time, but there wasn't this uncertainty of, ‘What now?’

Nonetheless, the question of overall benefit remains unanswered. Although nurses have had positive experiences, some question whether this technology is really what they need to improve their overall work situation and patient care.

## Discussion

### Principal Findings

This study used a parallel mixed methods triangulation design to explore nurses’ perspectives and experiences of a BES in an acute care hospital. The BES was implemented in 3 wards in a university medical center. Data collection included standardized online surveys completed by nurses at two time points: before (P0) and after (P1) the implementation of the system. In addition, FGs were conducted at both time points, using a semistructured interview guide. Retrospective prevalence surveys anonymously documented patient-related data, including cognitive impairment, tendencies to get out of bed, and AEs such as falls, over 24-hour periods.

The results clearly show the emotional and psychological strain nurses experience when caring for and supporting patients with cognitive impairment. The reported workload was significantly higher than in a survey on the care of people with dementia in hospitals by Isfort et al [23]. In the survey, 66.9% of participants reported high levels of distress when patients with cognitive impairment were allowed to leave the ward unnoticed [23]. The same question in our survey resulted in over 90% agreement. The other statements also showed significantly higher reported distress. Although this is not a representative cross-section, the results are worth discussing. One explanation could be the different target group of respondents. Isfort et al [23] surveyed senior nurses who were at least no longer working full-time “at the bedside.” The respondents in the current study were predominantly nurses working in direct patient care, so a greater potential for distress might be expected in this context. Another explanation may be that the participating units generally have an above-average number of patients with cognitive impairment. The nurses’ general attitude and acceptance of IT and communication technology are positive. This is also consistent with the results of an online survey conducted by the German Center for Quality in Care [30].

Nearly half of nurses reported that the BES made their daily work easier or much easier. Depending on the situation, it can increase both patient safety and nurses’ sense of safety. A BES can be a tool, but, like all technological innovations in highly complex environments, it does not solve complex, interrelated problems [12,31]. A BES can provide a sense of safety and increased security in selected situations, individually adapted to the setting and the patients. Looking at the complete cases, the experiences with the BES deviate negatively from the expectations. For example, the positive effect in dealing with patients with cognitive impairment, which 93% (13/14) of participants expected before using the BES, was observed by only 64% (9/14) of the participants after using the BES. This finding suggests that the experience of using the BES did not live up to previous expectations; helpfulness fell short of expectations. One way to deal with this is to manage expectations in advance (also on the part of the manufacturer) [32]. Whether the final perceived usefulness is sufficient for further use must be decided based on the fit between individual needs and benefits [33]. An improvement in perceived usefulness could possibly be achieved by implementing differentiated information mechanisms through the call system.

The most common AEs are getting patients out of bed and falls. A total of 66.9% (P0) and 59.9% (P1) of the respondents reported them as very stressful. These results are consistent with the findings of Isfort et al [23], who showed that these two events are the most stressful factors for nurses. Comparing the results of the cognitive impairment prevalence survey and AEs in the 2 phases, the rate of AEs per patient day decreased by a factor of 0.6. However, there were significant differences between the wards, with sometimes conflicting results. One explanation could be that the clinical conditions and comorbidities of the patients differ, which could lead to differences in the ability to use the BES very accurately.

Bed-leaving tendencies do not stand alone, but must always be evaluated in the context of other individual patient behaviors as well as the conditions of the particular ward. Focusing on a specific tool necessarily takes into account only a small part of the picture, which does not exist in this form for the nurses participating in the FGs. The situations described in the discussions show the complexity of supporting nurses in dealing with patients with cognitive impairment. Previous studies have predominantly addressed the efficacy and effectiveness of BES in terms of reducing falls or fall rates [15,34,35]. As a result, the primary focus of research has been on evaluating the functionality of the technology. However, there are many other factors that determine whether a technology will be successfully used and adopted over time in a particular setting [12,31]. This study provides a broader picture of the potential benefits of BES in the care and support of patients with cognitive impairments from a nurse’s perspective.

The analysis of the FGs shows a generally open and pragmatic attitude toward technology in nursing. The benefits of BES from the nurses’ point of view can be, for example, physical relief (eg, fewer rounds at night), psychological relief (eg, a greater sense of security), or an improvement in the quality of care (eg, avoidance of patient injury). At the same time, it is important to avoid new challenges, such as high alarm rates and limited response options, which can lead to alarm fatigue. In light of the survey results showing high expectations of the benefits of the BES and low experience of the benefits, the FGs showed that the high expectations were related to the reduction of falls. In the nurses’ perception, this goal was not fully achieved. However, it helped to cope with the consequences and was therefore helpful in reducing stress. The FGs show that the BES is best suited for use at night and with patients at risk of falling and with mild cognitive impairment. This specification of use scenarios makes it possible to obtain benefits in the form of fall prevention or rapid response to falls without being overloaded by too many alarms. Our results suggest that the effectiveness of the intervention appears to be strongly influenced by several factors, such as the individual condition of the patient, the staff-to-patient ratio, and the timing of use.

### Strengths and Limitations

This paper discusses the implementation of a specific BES. Due to the different systems and implementation aspects, it is not possible to generalize the results. The sample size, 30 people in P0, 24 in P1 and 15 complete cases, is a limitation for complex statistical procedures. However, the combination of quantitative and qualitative methods provides a comprehensive perspective and deeper insights into the research topic. We collected data for the prevalence survey using documentation forms at around 2 PM, referring to the previous 24-hour period. Overlaps occurred on 3 days per ward (study start, transition between study phases, and study end), but we ensured that patient and case counts were accurate to the day. The heterogeneous patient population, with complex and fluctuating symptom patterns and congruent but not equivalent wards, introduces potential bias in the evaluation of the prevalence survey. The significant reduction in the AE rate suggests that the use of the BES may be beneficial, although it does not allow causal conclusions. We must remember that leaving the bed unattended was also considered an AE. An increase in the AE rate could indicate a reduction in underreporting, paradoxically a desirable outcome. A more precise numerical presentation of the different AEs would have been helpful. Another limitation is the different conditions of use of the technology in different ward designs, which limits the direct applicability of the results to other wards. Since the focus of this study was to capture the perspectives of nursing staff specifically, the diversity of participants was limited by the study design. Future research could, however, consider including a broader range of health care professionals or expanding the diversity of nursing staff to explore how different perspectives, such as those from interdisciplinary teams, may influence the outcomes of BES implementation. Due to recruitment difficulties, we conducted a postintervention FG with only 2 participants, which may have changed the dynamics of the discussion, making it more like a dyadic interview. There are some similarities and differences between these methods [36]. In our case, each group discussion maintained the same thematic focus and similar dynamics.

### Conclusions

Our results indicate that while the BES provides some assistance in managing patients with cognitive impairment, its impact is limited to specific scenarios and does not significantly reduce nurses’ workload or strain. In addition, our study highlights the importance of managing expectations regarding BES performance. Setting realistic expectations for both manufacturers and end users is essential to bridge the gap between expected and experienced benefits. To better evaluate the benefits of BES for nurses and increase the likelihood of successful long-term implementation, future studies should address not only objectively measurable changes in patient care but also subjective perspectives, structural factors, and technical specifications. Technical improvements, such as differentiated information mechanisms through the call system, could enhance its perceived usefulness and help reduce alarm fatigue. By examining these factors, a more comprehensive understanding of the BES’s impact can be achieved, supporting its appropriate implementation in acute care settings. Finally, these findings emphasize the importance of integrating technology like the BES through close collaboration with nursing staff. Such collaboration is crucial for driving effective health care innovation and ensuring that new technologies meet the needs of both patients and nurses.

## Appendix

Multimedia Appendix 1 Self-assessment of general experience and acceptance (minimum 1, maximum 10).

## Abbreviations

AE: adverse event
BES: bed-exit information system
FG: focus group
GRAMMS: Good Reporting of a Mixed Methods Study
