# Supplementary figures and images for: Nurses’ Perspectives and Experiences of Using a Bed-Exit Information System in an Acute Hospital Setting: Mixed Methods Study
Source: JMIR Form Res. 2025 Feb 5;9:e64444. doi: 10.2196/64444 (PMC11840387; doi:10.2196/64444)

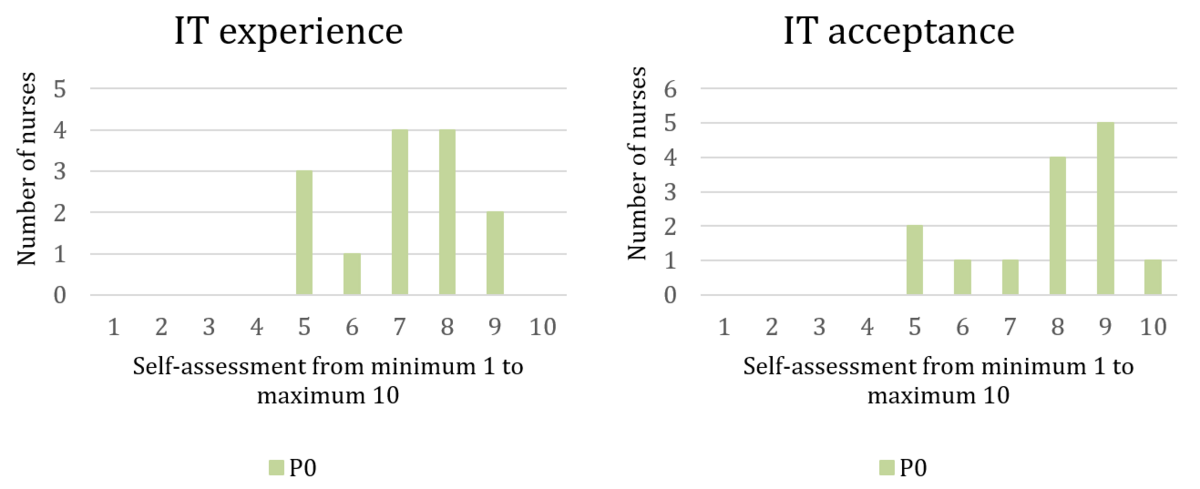

Supplement: Multimedia Appendix 1 [file formative_v9i1e64444_app1.png]
